# Epigenetic Modifications in Tumor-Associated Macrophages: A New Perspective for an Old Foe

**DOI:** 10.3389/fimmu.2022.836223

**Published:** 2022-01-24

**Authors:** Yuqin Niu, Jianxiang Chen, Yiting Qiao

**Affiliations:** ^1^ The First Affiliated Hospital, School of Medicine, Shihezi University, Shihezi, China; ^2^ School of Pharmacy, Department of Hepatology, the Affiliated Hospital of Hangzhou Normal University, Hangzhou Normal University, Hangzhou, China; ^3^ Division of Hepatobiliary and Pancreatic Surgery, Department of Surgery, The First Affiliated Hospital, Zhejiang University School of Medicine, Hangzhou, China; ^4^ NHC Key Laboratory of Combined Multi-organ Transplantation, Hangzhou, China; ^5^ Jinan Microecological Biomedicine Shandong Laboratory, Jinan, China

**Keywords:** epigenetic modification, tumor associated macrophage (TAM), histone modification, tumor microenvironment, methylation, acetylation, chromatin remodeling, lncRNA

## Abstract

Tumorigenesis is frequently accompanied by chronic inflammation, and the tumor microenvironment (TME) can be considered an ecosystem that consists of tumor cells, endotheliocytes, fibroblasts, immune cells and acellular components such as extracellular matrix. For tumor cells, their survival advantages are dependent on both genetic and epigenetic alterations, while other cells mainly present epigenetic modifications. Macrophages are the most plastic type of immune cells and undergo diverse epigenetic alterations in the TME. Some of these epigenetic modifications mitigate against cancer progression, and others accelerate this process. Due to the complex roles of macrophages in the TME, it is urgent to understand their epigenetic modifications associated with the TME. Here, we mainly summarize recent findings on TME-associated epigenetic alterations of tumor-associated macrophages (TAMs), including DNA methylation, posttranslational modifications of histone proteins, chromatin remodeling, and noncoding RNA-mediated epigenetic regulation. At the end of this review, we also discuss the translational potential of these epigenetic modifications for developing novel cancer therapies targeting TAMs.

## Macrophages and Tumor-Associated Macrophages

Macrophages are ubiquitously distributed mononuclear phagocytes originating from either hematopoietic stem cells or yolk sac-derived erythromyeloid progenitors (1). In the dermis, intestine, and peritoneum, macrophages mainly differentiate from bone marrow-derived monocytes. In other organs, such as the brain, lung and liver, a considerable portion of macrophage are represented by self-sustainable populations of tissue-resident macrophages originating from embryonic progenitors (2). Macrophages play important roles in both innate and adaptive immune responses by engulfing foreign substances, presenting antigens, and communicating intercellularly with other components of the immune system *via* surface proteins and secreted cytokines (3). Apart from their immunologic functions, macrophages are actively involved in diverse biological processes, such as angiogenesis, skeletal development, wound healing and malignant transformation (4).

The functional diversification of macrophages is largely attributed to their phenotypic plasticity (5). The process through which macrophages obtain distinctive phenotypic features in response to certain stimuli within their niche is called “polarization”. For the convenience of study, macrophages classically activated by proinflammatory signals such as interferons, granulocyte macrophage colony-stimulating factor (GM-CSF), and lipopolysaccharide (LPS) are defined as “M1 macrophages”, and macrophages alternatively activated by anti-inflammatory factors such interleukin-4 (IL-4), interleukin-10 (IL-10) and transforming growth factor-β (TGFβ) are defined as “M2 macrophages”. Correspondingly, the process through which macrophages acquire the phenotype of M1 macrophages such as the production of tumor necrosis factor (TNF), IL-1, and IL-12 is referred to as “M1-type polarization” or “M1 polarization”, while the process through which macrophages acquire the phenotype of M2 macrophages such as the expression of scavenger receptors and high indoleamine 2,3-dioxygenase activity is referred to as “M2-type polarization” or “M2 polarization” (some scientists also use the term “alternative activation”) (6). The term “M1 or M2 polarization” defines the status of macrophages according to the stimulus, while the term “classical or alternative activation” defines the status based on biological consequences. Even though these classification systems are often used interchangeably these days, the intensive study conducted by Marco Orecchioni et al. still revealed some inequivalence which merits some attention (7).

In biological processes other than the activation and resolution of immune responses, the status of macrophages may lie in between those extremes, and retain a certain level of plasticity towards either M1 or M2 polarization in response to environmental signals (8). Tissue-specific resident macrophages are key players for tissue homeostasis by forming either trophic or cytocidal interactions with neighboring cells, remodeling extracellular matrix, clearing dying cells, and secreting bioactive molecule such as cytokine, chemokines, growth factors, enzymes, arachidonate, oxygen, nitrogenderived metabolites, microvesicles and exosomes (9). The imbalance of macrophage functions has been documented to play a role in the many disease such as rheumatoid arthritis, chronic lung diseases and liver disorders (10–13). The interactions between microenvironment and resident macrophages have been reviewed by many researchers (14–16). Therefore, we would not expand this topic here.

Scientists have long noticed that M1-polarized macrophages can effectively phagocytose tumor cells by recognizing elevated phosphatidylserine levels in the outer membrane leaflet of malignant cells *in vitro (* 17). However, later studies revealed that although macrophages ranked first among tumor-infiltrating immune cells, their tumoricidal activities were often poor *in vivo (* 18). Over the same period, oncologists started to consider solid tumors as a unique microenvironment characterized by uncontrollable proliferation, dysfunctional vascularization, abnormal extracellular matrix structure, chaotic cell infiltration and harsh chemical conditions resulting from hypoxia and altered metabolism (19). Moreover, the tumor microenvironment (TME) profoundly remodels the component and functions of immune cells (20, 21). For example, more suppressive immune cells, such as myeloid-derived suppressor cells and regulatory T cells, are enriched in TME, while cytotoxic T cells and natural killer cells become highly exhausted (22, 23). Abundant evidence has demonstrated that such TME preferentially leads macrophages to undergo M2 polarization, and in turn the altered macrophages reinforce the TME in favor of more malignant growth (24). Therefore, TME-infiltrating macrophages are specifically referred to as “tumor-associated macrophages (TAMs)” since they are profoundly shaped by this disease and extensively involved in the disease progression. For example, both hypoxia and tumor cell-derived lactic acid could induce infiltrating macrophages to express more M2 marker CD206, vascular endothelial growth factor (VEGF) and Arginase I (25). VEGF-induced angiogenesis is crucial for a sustained supply of nutrients supporting tumor growth, while Arginase I catalyze the hydrolysis of L-arginine to deplete this key nutrient required for the proliferation of T cells and natural killer cells (26). Similarly, Itsaso Montalbán Del Barrio et al. showed that ovarian cancer cell-derived adenosine induced TAMs to express more ectonucleotidases CD39 and CD73, which further increased the concentration of immune-suppressive adenosine in the TME (27). The crucial roles of TAMs in tumor progression, metastasis, and resistance to therapy are perennial issues for research articles and reviews (28–32). Recently, Kaiyue Wu et al. published an instructive review regarding the subpopulations, functions and novel research techniques for understanding TAM-TME interaction (33). Therapies aimed at eliminating TAMs or “re-educating” them from M2 to M1 polarization have achieved remarkable success in both preclinical and clinical studies, and this topic was recently reviewed by Jiawei Zhou et al. (34).

TAMs undergo intense local proliferation in response to TME-required macrophage colony stimulating factor (MCSF), and the progenies maintain M2-polarized phenotypes (35). Consistent stimuli from the TME contribute to the directional transcription profiles of TAMs. The involvement in the phenotypic TAM remodeling of critical TFs such as hypoxia-inducible factor 1-alpha (HIF1α) and signal transducer and activator of transcription 3 (STAT3) has been thoroughly reviewed by many researchers (36, 37). Apart from TF theory which builds a real-time correlation between environmental signals and gene expression patterns for macrophages, scientists have long noticed the existence of memory-like behaviors. For example, macrophages trigger faster activation in response to recurrent signals, which is mediated by epigenetic modifications of latent enhancers in the genome of macrophages (38). In this review, we summarize the recent findings regarding the epigenetic modifications involved in the education of TAMs by the TME.

## Epigenetic Modifications Associated With Macrophage Education by the TME

Deoxyribonucleic acid (DNA) is the main substance controlling the inheritance of biological traits in every organism except prions and some viruses, but it is fascinating that embryonic cells harboring the same set of genes can produce all different types of cells with divergent phenotypes in a multicellular organism without alterations of DNA sequences. Therefore, the term “epigenetics” was proposed by Conrad Waddington in the 1940s to describe the research aimed at deciphering the mechanisms by which the same repertoire of genes could produce various phenotypes in specific niches during the development of metazoans (39). Later, the concept of epigenetics was adopted by a broader range of principles and redefined to include studies on both covalent and noncovalent modifications of DNA and histone proteins as well as the overall modifications of chromatin structures in any biological or pathological process (40). For example, the methylation of DNA, the posttranslational modifications of histone proteins, chromatin remodeling, and the influences of noncoding RNAs on nucleosome structure all belong to the research field of epigenetics. These epigenetic modifications convert environmental signals into distinct portfolios of accessible genes, thus facilitating a limited number of transcription factors (TFs) to produce more divergent transcriptional profiles. Concurrently, environmental signals could edit epigenetic signatures at the expression level and/or the biological activity of enzymes and regulatory factors involved in epigenetic modifications. Such an intertwined relationship between inheritable epigenetic modifications and the consensus DNA/RNA/protein central dogma laid the theological foundation for the study of environmental adaptation at the cellular level.

Tissue macrophages exhibit amazing variance in cell morphology and functions within different organs. The expression of PU.1, the master macrophage regulator, initiates epigenetic lineage determination during which PU.1 preferentially binds to tissue-specific TFs to produce tissue-specific macrophages (41). These TFs includes Sall1, RUNX, GATA6, PPAR-г, and Spi-C for microglia, intestinal macrophages, peritoneal macrophages, alveolar macrophages and splenic macrophages, respectively (42). The expression of these tissue-specific TFs, as well as their binding preference, are profoundly dictated by epigenetic modification under the influences of microenvironment (43, 44). The landscape of epigenetic modification in some tissue macrophages has been thoroughly studied through high-throughput sequencing techniques, which provides a textbook example for the study of environment-driven epigenetic modification (45).

In the context of oncology research, epigenetic modification is one of the major mechanisms by which the TME affects behaviors of infiltrating cells, such as fibroblasts, vascular endothelial cells, lymphocytes and tumor cells themselves (46, 47). The involvement of dysregulated epigenetic modification in the growth and metastasis of cancer cells has also been heavily reviewed (48–50). A study comparing the epigenetic reprogramming patterns in paired primary and distant metastasis of pancreatic ductal adenocarcinoma specimens revealed that global changes were targeted to thousands of large chromatin domains across the genome that collectively specified malignant traits (51). This study marks that we have started to understand the landscape of cancer epigenetics at both spatial and temporal dimensions (52). Probably due to availability of cell line models and clinical samples, the mechanisms and biological effects of epigenetic dysregulation in cancer cells and cancer-related fibroblasts (CAFs) are more thoroughly studied and reviewed (53–56), compared to the other types of cells present in TME. The critical components involved in dysregulated epigenetic signaling network might be shared among different types of cells, but the biological consequences are highly cell-type specific (57). The overall epigenetic landscape of macrophages and other tumor-infiltration immune cells has been reviewed by several scientists recently (58–60). In this review, we would mainly discuss experimental studies as well as the potential applications regarding the epigenetic modifications associated with TAMs, which might inspire more basic and translational researches to investigate the epigenetic modifications of macrophages in malignant transformation and cancer management.

## DNA Methylation

**Figure 1:**
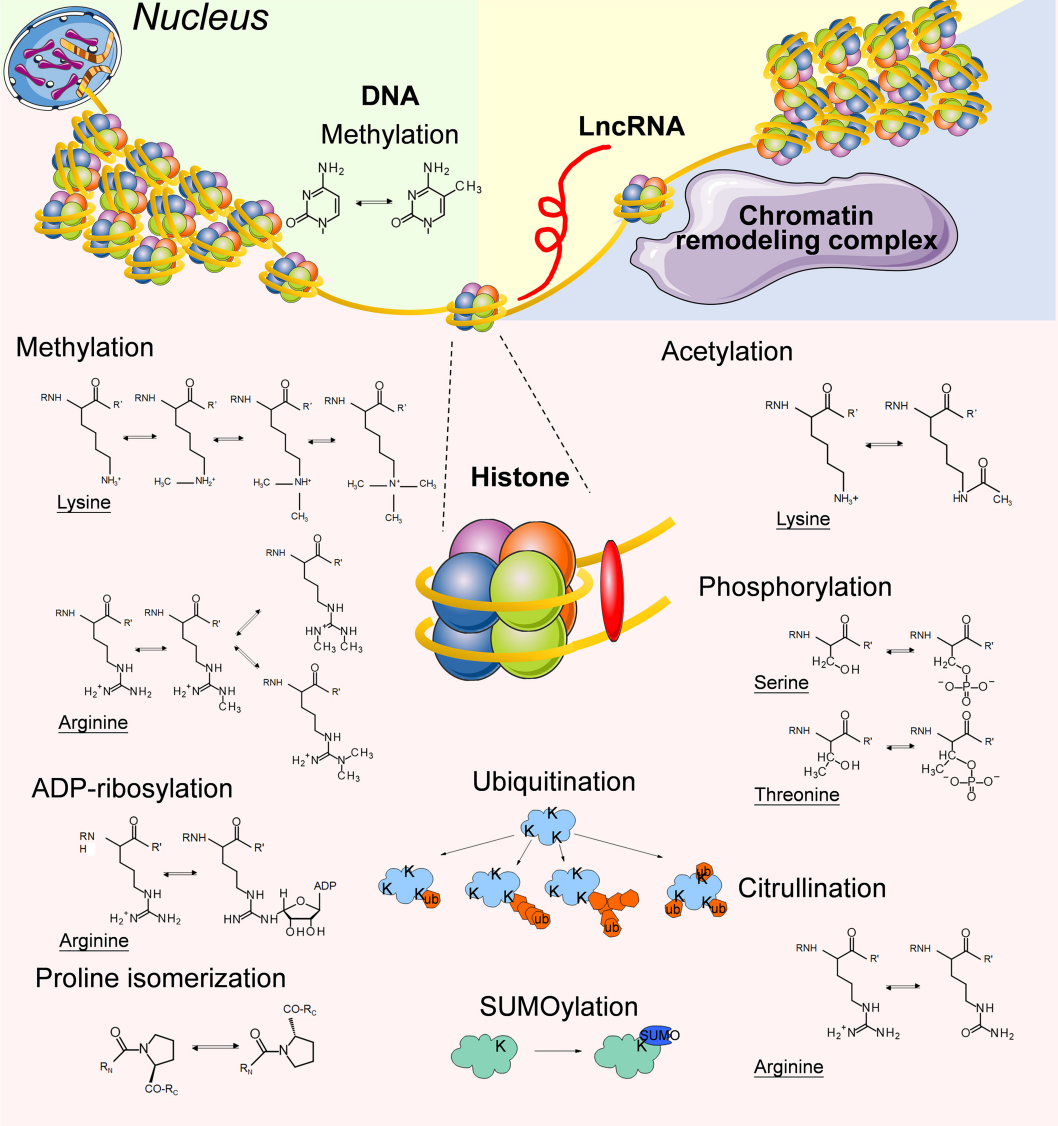
Summary of the typical epigenetic modifications on genomic DNA, Histone proteins and LncRNAs.

In DNA methylation, methyl groups are covalently added to DNA bases, most frequently the cytosine of CpG dinucleotides (**Figure 1**), and the methylation of CpG islands in promoters typically leads to the silencing of gene expression. In mammals, cytosine methylation is mediated by DNA methyltransferases (DNMTs) including DNMT1, DNMT2, DNMT3A, DNMT3B, and DNMT3L, while the conversion of methylcytosine to 5-hydroxymethylcytosine is catalyzed by Tet methylcytosine dioxygenases (TETs) including TET1, TET2 and TET3 (61).

**Figure 2:**
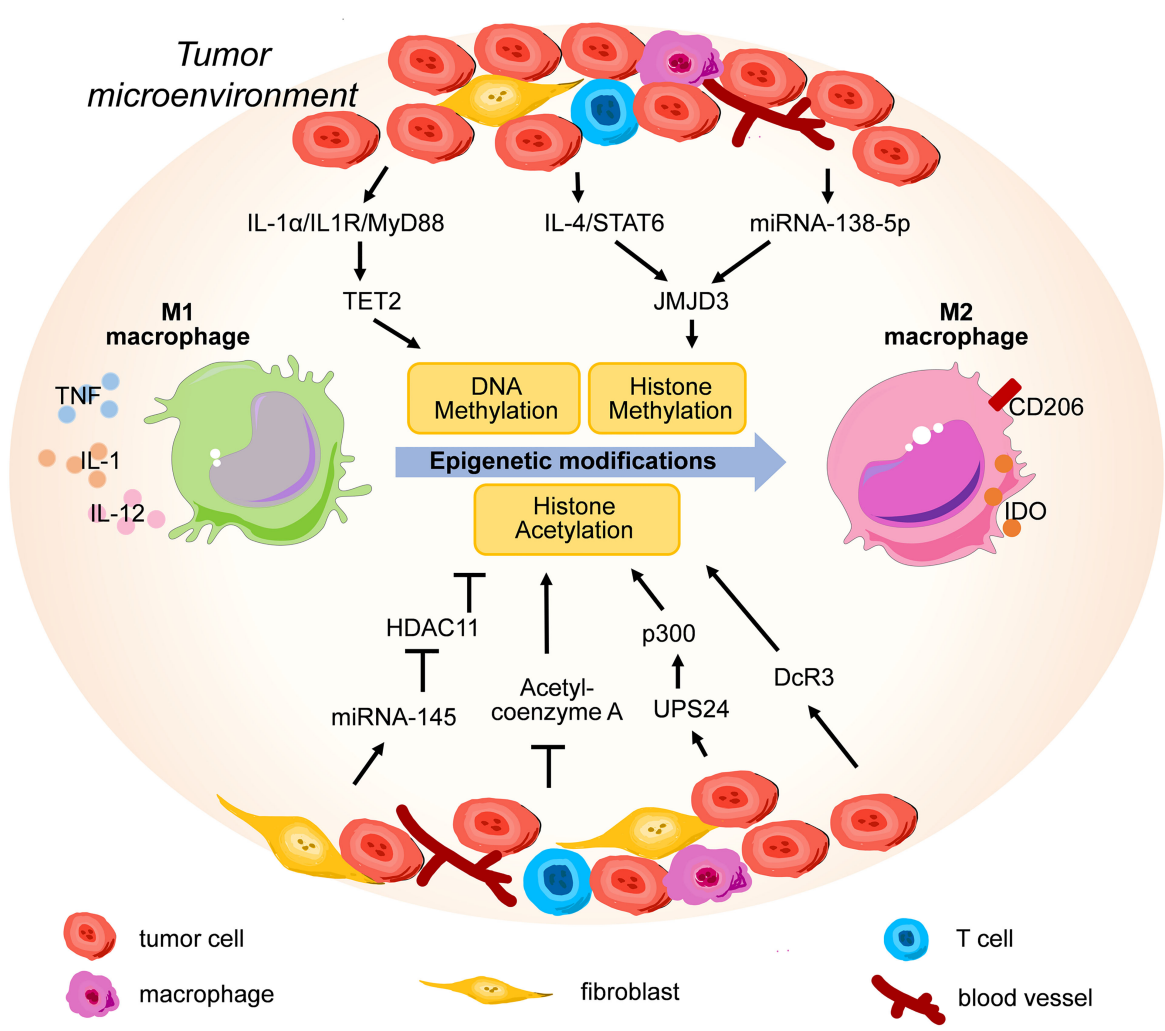
The representative epigenetic signaling regulatory factors and pathways involved in modulating M2 polarization in tumor microenvironment.

TET2 is one of the most highly expressed Tet enzymes in murine macrophages, and it is important for the resolution of LPS-induced inflammation by restraining IL-1β, IL-6, ARG1, and chemokine expression at the late phase of the immune response (62, 63) (**Figure 2**). Wen Pan et al. discovered that TET2 expression was enhanced in an IL1R/MyD88 pathway-dependent manner in TAMs isolated from both murine and human melanoma specimens, and myeloid-specific ablation of *Tet2* led to suppressed melanoma growth *in vivo* by modulating the gene expression program from an immunosuppressive status into a proinflammatory status in TAMs (64). Tumor cell-derived IL-1α has been linked with higher metastasis in lung cancer and breast cancer, indicating potential therapeutic value for blocking the IL-1α/IL1R/MyD88/TET2 axis (65, 66). Some scientists have noticed that TET2 might be the intersection between cancer and immunity (67, 68).

Studies focusing on the role of DNMTs in TAMs are sparse. Therefore, we could only speculate based on data obtained in other biological systems. DNMT1 has been shown to play crucial roles in M1 activation by suppressing the expression of Krüppel-like factor 4 (KLF4) and suppressor of cytokine signaling 1 (SOCS1), and DNMT1 overexpression enhances the secretion of proinflammatory cytokines such as TNFα and IL-6 in RAW264.7 cells (69, 70). Similarly, a high level of DNMT3B promotes M1 polarization by methylating the promoter region of peroxisome proliferator activated receptor γ (*Pparg*) in murine adipose tissue macrophages, and *Dnmt3b* silencing induces M2 polarization in RAW264.7 cells (71). All these studies link the activation of DNMTs with M1 polarization in macrophages. Recently, a study of pancreatic cancer reported that the direct cell-cell contact between cancer cells and macrophages would lead to a suppressed glucose metabolic status by changing the DNA methylation pattern of oxidative phosphorylation-associated genes in M1 macrophages, but not in M2 macrophages, and this cell-cell interaction could be blocked by the pre-treatment of DNMTi (72). Mechanistic study showed that Glycoprotein A Repetitions Predominant (GARP) on the membrane of macrophage and integrin αV/β8 on the membrane of the cancer cell played crucial roles in this process. However, the detailed signaling pathway connecting cell membrane signals with DNMTs activity remains unsolved.

Analyses of bulk tissues have identified DNMT3B overexpression in many types of solid tumors such as breast and colorectal cancers (73, 74), and more precise analyses specifically focusing on DNMT expression and DNA methylation status in TAMs are urgently required. DNMT inhibitors (DNMTi) such as 5-Aza-2’ deoxycytidine (5-Aza-dC, decitabine, DAC) and 5-azacytidine (5-AC) can sensitize cancer cells to various therapeutics (75, 76), although their influence on TAMs is still controversial. For example, *in vivo* treatment with 5-AC led to decreased M2 macrophages and increased M1 macrophages through the enhancement of type I IFN signaling in the TME in a murine ovarian cancer model (77, 78), while *in vitro* treatment with DNMTi resulted in significantly attenuated proinflammatory functions in RAW264.7 cells and isolated primary murine peritoneal macrophages (79, 80). Recently, a preliminary RNA-sequencing study of DAC-treated murine pancreatic ductal adenocarcinoma tissues revealed increased expression of *Chi3l3*, reflecting an increase in a subset of tumor-infiltrating M2-polarized macrophages (81). A lot of DNMTi-associated work was conducted in macrophage cell lines, murine models and atherosclerosis-associated macrophages, so there is an urgent need for more direct evidence to conclude the influence of DNMTi therapies on TAMs in more clinically relevant models.

In addition to the cytosine of DNA, the adenine of RNA can also be methylated to form N6-methyladenosine (m6A), which has a remarkable influence on various biological behaviors of RNA such as splicing, stability and translation (82). Recently several studies have focused on the role of RNA m6A in macrophage activation and cancer-associated reprogramming (83, 84). Scientists have indicated that RNA m6A may be a form of epigenetic regulation. However, in this review, we mainly focus on narrowly defined epigenetic modifications related to chromatin.

## Histone Methylation

Histones are central players that maintain the chromatin structure. Approximately 147 base pairs of DNAs are wrapped around an octamer of histones (2 copies each of H3, H4, H2A and H2B) to form the nucleosome core particle, while histone H1 interacts with DNA of variable length and links adjacent nucleosome cores to further compact the chromatin (85). Histone methylation means the modification of arginines and lysines by the addition of 1, 2, or 3 methyl groups to histone proteins (86) (**Figure 1**). Histone methylation influences the compaction of chromatin and accessibility to TFs as well as other regulatory protein complexes. Therefore, it plays an important role in transcriptional activation and repression. There are 2 types of histone methyltransferases (HMTs): histone lysine N-methyltransferases and histone arginine N-methyltransferases (87). Histone demethylation is mediated by histone demethyltransferases (HDMs), including lysine-specific demethylase, Jumonji domain-containing hydroxylases (JMJDs) and peptidyl arginine deiminases. The coexistence of redundant HMTs and HDMs indicates that histone methylation can be reversely regulated by environmental signals (88).

Irina Tikhanovich et al. discovered that HMT protein arginine methyltransferase 1 (PRMT1) positively regulated *Pparg* gene expression through histone H4R3me2a methylation at the *Pparg* promoter in murine macrophages, and PPARγ was one of the key transcription factors promoting M2 polarization (89). Following this discovery, Jie Zhao et al. showed that the PRMT1/IL-6/STAT3 axis promoted alcohol-associated HCC progression by inducing M2 polarization in mice fed with Lieber-DeCarli alcohol liquid diet, and PRMT1 expression was correlated with STAT3 activation in TAMs in human HCC specimens (90). These results suggest that PRMT1 is a potential therapeutic target in TAMs for alcohol-associated HCC immunotherapy. Recently, Xiuling Wang et al. reported that G9a, another HMT, could promote lipid-induced M1 macrophage polarization by negatively regulating CD36 (91). However, the involvement of G9a in the TME-TAM interaction requires further investigation. These examples also highlight the importance of specificity during the development of antagonists and agonists for each HMT because different HMTs might play opposite roles in M1/M2 polarization.

Evidences have shown that the expression level of the H3K27 demethylase JMJD3 could be influenced by cytokines and tumor-derived exosomes present in the TME, and a high level of JMJD3 contributes to M2 polarization. For example, Makoto Ishii et al. reported that the activation of the IL-4/STAT6 signaling axis increased the expression level of JMJD3, which decreased the suppressive H3K27 methylation at the promoter of genes related to M2 polarization (**Figure 2**), including *Chi3l3*, *Retnla*, *Arg1*, *Nos2* and *Irf4* using a schistosoma mansoni egg-challenged mouse mode *in vivo (* 92, 93). Concurrently, an *in vitro* study also demonstrated that IFNγ could also increase JMJD3 mRNA levels in human monocyte-derived macrophages (94). Recently, Jing Xun et al. discovered that breast cancer cells induced TAMs to express more JMJD3 by secreting exosomes containing microRNA-138-5p (**Figure 2**), thus enhancing M2 polarization in TAMs (95).

However, great caution must be taken when attempting to predict the biological function of one HMT/HDM based on the other because the summation of direct and indirect influences of these epigenetic regulators on macrophage polarization could be dramatically diverse, due to different target preferences and modification sites. For example, SMYD3 and SET7/9, both of which are HMTs, induce activating histone codes on the promoters of *S100A9* and *S100A12* in response to glucose, thus promoting M1 polarization (96), while SMYD5-mediated H4K20 trimethylation and SMYD2-mediated H3K36 dimethylation function as repressive checkpoints for the expression of TLR4 target genes in macrophages (97, 98). Similarly, both SETD4 and ASH1L are H3K4 methyltransferases. SETD4 positively regulates IL-6 and TNFα expression in TLR agonist-stimulated macrophages by directly activating H3K4 methylation (99), while ASH1L enhances the expression of tumor necrosis factor alpha-induced protein 3 (*Tnfaip3*) through the induction of H3K4 methylation at the *Tnfaip3* promoter, thus suppressing IL-6 and TNFα production in TLR-triggered macrophages (100). The opposite biological effects for these two H3K4 methyltransferases in response to the same stimulus suggest a high level of complexity for the functional interplay between HMT/HDMs and transcriptional regulation. However, most of our current knowledge is obtained from *in vitro* experiments deprived of TME signals, which might lead to biased conclusions. Thus, the utilization of both macrophage-specific transgenic mice and tumor models is required to precisely and comprehensively understand the biological roles of these HMT/HDMs in the process of tumor progression.

## Histone Acetylation

Histone acetylation and deacetylation are catalyzed by histone acetyltransferases (HATs) and histone deacetylases (HDACs) at lysine residues along histone tails (101) (**Figure 1**). Histone acetylation generally indicates transcriptional activation, and histone deacetylation often correlates with repressed transcription (102).

Many studies have directly or indirectly demonstrated that the functions of macrophages could be finetuned *via* histone acetylation profiles by signaling molecules present in the TME (**Figure 2**). For example, Haruka Shinohara et al. reported that colorectal cancer cells induced M2 polarization by exosome-mediated secretion of miRNA-145 to downregulate HDAC11 expression in TAMs, thus leading to significant enlargement of the tumor volumes in DLD-1 cell-xenografted mice (103). In addition to exosomes, cells in the TME constantly compete for nutrients such as glucose and oxygen, and redirect cellular metabolism from oxidative respiration to anaerobic glycolysis, which reduces the production of acetyl-coenzyme A (104). Mario A. Lauterbach et al. discovered that macrophages increased glycolysis and tricarboxylic acid cycle volume to generate more acetyl-coenzyme A from glucose upon TLR4 activation, thus leading to augmented histone acetylation which facilitated the transcription of LPS-inducible gene sets contributing to M1 polarization (105). This study connects the aerobic metabolism pathway with proinflammatory polarization *via* histone acetylation, which might partially explain the phenotypic shift from M1 to M2 for TAMs during malignant transformation in a hypoxic TME.

The production of IL-6, a key interleukin inducing M2 polarization, is frequently regulated by histone acetylation in its promoter. For example, Yi-Chang Wang et al. discovered that leukocytes infiltrating tumors expressed higher ubiquitin-specific peptidase 24 (USP24) levels than surrounding tumor cells in lung cancer patient specimens, and USP24 increased the level of histone H3 acetylation in the promoters of NFKB1 and IL-6 by stabilizing HAT p300, thereby increasing the expression of these genes in M2 macrophages to promote the progression of lung cancer (106). In another experimental condition, Lingli Hu et al. reported that sodium valproate, a widely used HDAC inhibitor, increased histone activation marks H3K4me3 and H3K9ac at *Il6* promoter regions in a murine paraquat-induced pulmonary fibrosis model (107).

Yung-Chi Chang et al. utilized an *in vitro* cell model, transgenic mouse model, and clinical specimens to demonstrate that decoy receptor 3 (DcR3) suppressed the expression of genes involved in MHC-II-dependent antigen presentation by inducing deacetylation of histones associated with the promoter of CIITA, the master regulator of MHC-II expression, which led to M2 polarization in TAMs (108). Later, the same group reported that xenograft growth and spreading were significantly enhanced by monocyte-specific DcR3 expression in CT26 mice colorectal cancer model, and DcR3-induced tumor growth was blocked by the HDAC inhibitor sodium valproate (109). Keratinocytes enhance DcR3 expression in response to epidermal growth factor (EGF), transforming growth factor α (TGFα) and TNFα in psoriasis patients (110), and lung fibroblasts increase DcR3 expression *via* the Akt/GSK-3β/NFATc1 signaling axis in contact with the collagen matrix in patients with idiopathic pulmonary fibrosis (111). However, whether these TME-associated factors could upregulate DcR3 expression in macrophages remains to be experimentally investigated.

## Other Histone Posttranslational Modifications

Similar to other proteins, histones undergo a variety of posttranslational modifications (PTMs) (112). We have discussed the involvement of methylation and acetylation, the two most frequently mentioned types of histone PTMs for epigenetic research, during the education of TAMs by TME in previous paragraphs. The roles of other histone PTMs in TAM/TME interactions are relatively less studied. Thus we summarized several studies regarding arginine citrullination, lysine ubiquitination, lysine SUMOylation, ADP-ribosylation, proline isomerization, and serine/threonine/tyrosine phosphorylation occurring in histones (**Figure 1**).

Protein phosphorylation means the addition of phosphate groups to serine, threonine, tyrosine, or sometimes histidine residues of proteins, thus creating a negative charge at the site of modification (113) (**Figure 1**). Protein phosphorylation is catalyzed by protein kinases, and its removal is mediated by protein phosphatases. The phosphorylation of histone H1 at multiple sites has been demonstrated as one of the prerequisite steps for gene induction *in vitro (* 114–117). Steven Z.Josefowicz et al. discovered that in LPS-stimulated mouse macrophages, mitogen- and stress-activated protein kinases (MSKs) phosphorylate histone H3 at serine 28, which directly promotes p300/CBP-dependent transcription (118). More interestingly, Sayantan Banerjee et al. reported that both the lack of transcription favorable histone phosphorylation at the IL-12 promoter and the abundance of ERK1/2-dependent histone phosphorylation at the IL-10 promoter led to the polarization of TAMs toward a more immunosuppressive form, although the mechanism underlying the ability of the TME to preferentially change the phosphorylation pattern of histones in TAMs has not been clarified (119).

Citrullination is the posttranslational conversion of peptidylarginine to peptidylcitrulline *via* the catalysis of calcium-dependent peptidylarginine deiminases (**Figure 1**), thus leading to changes in the positively charged arginine residue to an uncharged citrulline residue (120). The level of citrullinated histone H3 significantly increases after LPS stimulation in macrophages derived from U937 cells *in vitro*, as a result of the enhanced expression level of peptidylarginine deiminases (121). The involvement of histone H3 citrullination in epigenetic regulation or gene expression has not been thoroughly studied, but it has been demonstrated to be an important step in the formation of extracellular traps for immune cells (122).

Ubiquitination refers to the addition of a single ubiquitin molecule (monoubiquitination), or the conjugation of ubiquitin to preceding ubiquitin moieties (polyubiquitination) to lysine residues of proteins (123) (**Figure 1**). Ubiquitin is a 76 amino acid polypeptide, and ubiquitination leads to a dramatic change in protein conformation. Monoubiquitination tends to be considered a signal transduction event, while polyubiquitination is a typical recognition marker for 26S proteasomal proteases (124). Monoubiquitination of H2A at lysine 119 prevents the recruitment of SPT16 and SSRP1 at the transcriptional promoter region, and blocks RNA polymerase II release at the early stage of elongation, which mediates selective repression of a specific set of chemokine genes modulating migratory responses to TLR activation in macrophages, such as *Ccl5, Cxcl2*, and *Cxcl10 (* 125).

Similar to ubiquitination, SUMOylation means covalent ligation of small ubiquitin-related modifier (SUMO) groups, which are approximately 100 amino acids in length, to lysine residues of a protein (126) (**Figure 1**). Our current knowledge indicates that histone SUMOylation generally mediates gene silencing through recruitment of HDAC and heterochromatin protein 1 (127, 128), although its involvement in macrophage polarization has not been thoroughly investigated.

ADP-ribosylation refers to the transfer of an ADP-ribose moiety from NAD+ to amino acid residues, such as lysine, arginine, glutamate, aspartate, cysteine, phosphoserine, and asparagine, which is mediated by ADP-ribosyltransferases (129) (**Figure 1**). ADP-ribosylation increases the negative charge of the modified protein. Mono-ADP-ribosylation has been detected in all 4 core histones and the linker histone H1 in mammalian cells (130). Upon LPS stimulation, the enzymatic activity of chromatin-associated poly(ADP-ribose) polymerase 1 (PARP-1) increases, and the ADP-ribosylation modification of histones destabilizes histone-DNA interactions in the nucleosome, thus improving site accessibility at the gene loci of *Il1b*, *Mip2*, and *Csf2* in macrophages (131). The study of protein ADP-ribosylation modification in immunology and cancer has gained increasing attention in recent years, although the involvement of histone ADP-ribosylation in TAMs has not been thoroughly investigated yet (132).

Apart from covalent modification at different residues of histones, the conformational state of prolines in histones also plays a role in epigenetic regulation (**Figure 1**). For example, isomerization of prolines at the amino-terminal tail of histone H3 by proline isomerase FPR3 inhibits the methylation of H3K36 by SET2 (133).

## Chromatin Remodeling Enzymes

The fundamental subunit of chromatin is called the “nucleosome”, which is composed of DNA wrapped around the histone octamer, and then chromatin is further packaged into heterochromatin with a higher structure to reduce volume (134). Therefore, gene transcription requires alterations of compact chromatin structure, which allow the exposure of active DNA segments to transcription machinery under the action of ATP-dependent chromatin-remodeling enzymes (135, 136).

There are five major families of ATP-dependent chromatin-remodeling enzymes: SWI/SNF, ISWI, Nurd/Mi/CHD, SWR1, and INO80 (137). Their roles in macrophage polarization are quite diverse. For example, the catalytic BRG1/BRM subunits of the SWI/SNF class of ATP-dependent nucleosome remodeling complexes are consistently required for the activation of secondary response genes and primary response genes induced with delayed kinetics in LPS-stimulated macrophages, while a Mi-2β complex is selectively recruited along with SWI/SNF complexes to act antagonistically to limit the induction of these gene classes (138).

Systematic studies on the involvement of ATP-dependent chromatin-remodeling enzymes in TAMs are still in the very early stages. Ping-Chieh Chou et al. preliminarily reported an interplay between tumor-secreted IGFBP2 and ATP-dependent chromatin-remodeling enzyme INO80 in pancreatic ductal adenocarcinoma (PDAC) as a conference poster and showed that PDAC tissues often secreted excessive Insulin-like growth factor binding protein-2 (IGFBP2) to pancreatic juice and serum, and tumor-secreted IGFBP2 directly regulated INO80 functions and inhibited MHC class II expression in macrophages (139). The mechanism underlying this interaction is unknown yet, although it likely occurs *via* direct protein-protein interactions (140).

## Noncoding RNA-Mediated Epigenetic Regulations

Noncoding RNAs are a special class of regulatory RNAs that are not translated into proteins (141). Noncoding RNAs that have approximately 200 nucleotides or more are called “long noncoding RNAs (lncRNAs)”, while the longest lncRNAs, which are much longer than 200 nucleotides, are referred to as “long intergenic non-coding RNA (lincRNA)” (142). LncRNAs are highly structured biomacromolecules capable of simultaneously interacting with proteins, DNA, and other RNA *via* different motifs within one molecule (143). Therefore, lncRNAs can guide epigenetic modulators catalyzing epigenetic modifications to specific regions of the genome in both *cis* and *trans* manners (144).

In macrophages, lincRNA-EPS restrains the expression of immune response genes by interacting with heterogeneous nuclear ribonucleoprotein L to induce the aggregation of nucleosomes *via* a CANACA motif located in its 3′ end, and genetic deletion of lincRNA-EPS leads to enhanced basal and TLR4-induced expression of immune response genes in macrophages (145). However, the mechanisms by which lincRNA-EPS specifically localizes to the genomic loci of target genes remain unsolved. Moreover, the regulatory mechanism for its expression and its association with TAM transformation also requires further investigation. Another lncRNA, CDKN2B-AS,1 has been shown to form an RNA-DNA triplex with the CDKN2B promoter and recruit EZH2 and CTCF to inhibit CDKN2B transcription by accelerating histone methylation in macrophages (146). A similar RNA-DNA triplex might also exist for lincRNA-EPS.

Whole-transcriptome analysis of macrophages stimulated with the synthetic TLR2 ligand Pam_3_CSK_4_ revealed that Pam_3_CSK_4_ treatment significantly increased the level of lincRNA-Cox2 in macrophages. Silencing of lincRNA-Cox2 led to attenuated Pam_3_CSK_4_-induced expression of TLR1 and IL-6 (147). Mechanistically, lincRNA-Cox2 is assembled into the SWI/SNF complex in macrophages after TLR ligand stimulation, and the lincRNA-Cox2/SWI/SNF complex can modulate the assembly of NFκB subunits to the SWI/SNF complex to induce the transactivation of late primary inflammatory response genes in response to microbial challenge (148). However, whether the formation of the lincRNA-Cox2/SWI/SNF complex is influenced by TME-associated signals remains to be investigated.

## Translational Potential of Macrophage-Associated Epigenetic Modifications and Perspective

The reversible polarization of macrophages is a double-edged sword for cancer therapy. On one hand, a wide variety of pharmaceutical interventions could be utilized to drive M1 polarization and achieve tumoricides (149–151). On the other hand, high plasticity correlates with a high chance of being adversely influenced by environmental factors once pharmacological interventions are withdrawn (152). Therefore, although macrophages are superior to T cells due to their low dependency on antigen specificity, the development of macrophage-based immune cell transfusion therapy still lags behind T cell-based immune cell transfusion therapy. In 2017, Cory M. Alvey et al. reported that the injection of bone marrow-derived macrophages pretreated with SIRPα blocking antibody exhibited intratumoral accumulation and phagocytotic activity for 1-2 weeks in mouse model, although these macrophages quickly differentiated toward nonphagocytic TAM phenotype and lost tumor-suppressive activity (153).

The application of epigenetic interventions to “lock” macrophages at the M1 status might be one potential solution for their low phenotypic stability in the TME. For example, the exogenous expression of several epigenetic regulators, such as DNMT1 and DNMT3B, has been previously mentioned to enhance the M1 polarization of macrophages, while the silencing of some other factors, such as TET2 and PRMT2, could retard M2 polarization. Nowadays we have powerful tools for targeted gene editing and controlled protein degradation such as CRISPR-Cas9 and PROTAC technologies (154, 155). It would be theoretical feasible and rational to evaluate the long-term behavior of macrophages pretreated with the combination of SIRPα blocking antibody and genetic manipulation of such genes, and both therapeutic effects and carcinogenic safety should be taken into consideration during the evaluation.

A recent work by Mengwen Zhang et al. demonstrated that the infusion of M1 macrophages alone led to increased distal metastasis in a murine orthotopic pancreatic cancer model in which endogenous macrophages had been depleted, since M1 macrophages would be transformed to TAMs once they infiltrated TME. However, the pretreatment of infused macrophages with DNMTi could suppress the metabolic functions of TAMs and significantly reduced metastasis (72). The underlying mechanism has been discussed in the section of “DNA methylation” previously in this review. Even though the long-term effects of DNMTi could not be evaluated due to the limitation of short experimental duration, this study is still strong evidence demonstrating the potential application of epigenetically reinforced macrophages in cancer therapy. Moreover, this report also inspires the research community to think out of the box of M1/M2 redirection, but to utilize the difference between M1 and M2 macrophages for differentiated strike.

Apart from transfusion therapy, systematic treatment targeting intratumoral macrophages *in situ* is also a feasible strategy. Targeting elements such as mannose, structured peptides, DNA aptamers, and antibodies could be used in combination with drug encapsulation methods, such as lipid polymers and other high molecular weight materials (156–159). Although relative enrichment of drugs intratumorally is achievable, leakiness is inevitable. Therefore, a crucial question to be answered is the influence of these pharmaceutical compounds on other cell compounds in tumors as well as in healthy organs. Compared with therapies targeting cytokines and immune checkpoints, therapies targeting epigenetic modifications are more prone to unexpected systematic effects since these signaling pathways are highly infrastructural. Therefore, it is urgent to conduct more *in vivo* evaluations in studies regarding epigenetic interventions targeting TAMs. On the other hand, therapeutic interventions targeting tumor cells would simultaneously hit TAMs, and the altered behaviors of TAMs might play key roles in resistance and relapse, which requires great attention.

Therapies targeting TME-TAM interactions would also be beneficial for epigenetically regulating macrophage functions. Technologies for developing humanized neutralizing antibodies against cytokines or receptors involved in M2 polarization-associated epigenetic modifications, such as IL-4 and IL-1α, are mature these days (160). The concentration of these agents could be further increased in TME with the help of nanotechnology since macromolecular drugs tend to concentrate in tumors with abnormal tumorous vessels (161). Generally, cytokines influence more than one kind of immune cells, so these neutralizing antibodies would have potent power in re-shaping tumor immune microenvironment (162, 163). Some other interventions aimed at suppress the functions of other cells in the TME might have concomitant effects on TAMs. For example, gefitinib, an EGFR inhibitor suppressing tumor growth and angiogenesis, was recently shown to inhibit the crosstalk between macrophages and cancer cells by blocking receptor interacting protein kinase 2 (RIPK2) (164). With an increasing community of oncologists realizing the importance of TAMs, more attention would be payed to investigate macrophage phenotypes during the evaluation of cancer therapies.

Recently, Rongchen Shi et al. reported that systemic administration of DNMTi DAC stimulated the activation of TAMs towards an M1-like phenotype in a murine peritoneal carcinomatosis of colorectal cancer model. Mechanistically, DAC bond ATP-binding cassette transporter A9 and induced cholesterol accumulation, which increased p65 phosphorylation and IL-6 expression in a DNMTi-independent manner (165). The universality of the mechanism identified in this study has yet to be tested with more experimental evidence in other models, as we have discussed several studies with different results in the section “DNA methylation” previously in this review. However, it is generally acknowledged that DNMTi therapies would improve immune microenvironment from the point of tumor infiltrating T cells by re-activating the expression of immunosurveillance-related genes in tumor cell, and the combination of DNMTi with immune therapies has exhibited some therapeutic benefits in several studies (81, 166–168). Understanding the biological behaviors of TAMs in these processes might further improve these therapies.

In summary, TAMs, as the intratumorally infiltrating immune cells with the largest number, represent a force to be considered for successful cancer therapy (169). The TME affects the functions of TAMs partially through epigenetic modifications, and the investigation of such intercellular communication would lead to the discovery of more promising therapeutic targets for cancer immunotherapy targeting macrophages.

## Author Contributions

YN and YQ is responsible for the collection, collation, and writing of the manuscript. JC and YQ are responsible for the concept development, revision, and review of the manuscript. All authors contributed to the article and approved the submitted version.

## Funding

This work was supported by funding from the Natural Science Foundation of Zhejiang Province (LR21H160001, LY21H160025), “Pioneer” and “Leading Goose” R&D Program of Zhejiang Province (2021C03G2153004), National Natural Science Foundation of China (81802338, 82072646, 81903143), Start-up Grant of HZNU (4125C5021820470) and Research Project of Jinan Microecological Biomedicine Shandong Laboratory (JNL-2022029C).

## Conflict of Interest

The authors declare that the research was conducted in the absence of any commercial or financial relationships that could be construed as a potential conflict of interest.

## Publisher’s Note

All claims expressed in this article are solely those of the authors and do not necessarily represent those of their affiliated organizations, or those of the publisher, the editors and the reviewers. Any product that may be evaluated in this article, or claim that may be made by its manufacturer, is not guaranteed or endorsed by the publisher.
